# TSLP directly impairs pulmonary Treg function: association with aberrant tolerogenic immunity in asthmatic airway

**DOI:** 10.1186/1710-1492-6-4

**Published:** 2010-03-15

**Authors:** Khoa D Nguyen, Christopher Vanichsarn, Kari C Nadeau

**Affiliations:** 1 Department of Pediatrics, Stanford University, Stanford, CA, USA

## Abstract

**Background:**

Even though thymic stromal lymphopoietin (TSLP) has been implicated in the development of allergic inflammation, its influence on immune tolerance mediated by regulatory T cells (Treg) have not been explored. We aimed to dissect the influence of TSLP on immunosuppressive activities of Treg and its potential consequences in human allergic asthma.

**Methods:**

*In vitro *culture system was utilized to study the effects of TSLP on human Treg. The functional competency of pulmonary Treg from a cohort of 15 allergic asthmatic, 15 healthy control, and 15 non-allergic asthmatic subjects was also evaluated by suppression assays and flow cytometric analysis.

**Results:**

Activated pulmonary Treg expressed TSLP-R and responded to TSLP-mediated activation of STAT5. TSLP directly and selectively impaired IL-10 production of Treg and inhibited their suppressive activity. In human allergic asthma, pulmonary Treg exhibited a significant decrease in suppressive activity and IL-10 production compared to healthy control and non-allergic asthmatic counterparts. These functional alterations were associated with elevated TSLP expression in bronchoaveolar lavage fluid (BAL) of allergic asthmatic subjects. Furthermore, allergic asthmatic BAL could suppress IL-10 production by healthy control pulmonary Treg in a TSLP-dependent manner.

**Conclusions:**

These results provide the first evidences for a direct role of TSLP in the regulation of suppressive activities of Treg. TSLP mediated inhibition of Treg function might present a novel pathologic mechanism to dampen tolerogenic immune responses in inflamed asthmatic airway.

## Background

Thymic stromal lymphopoietin (TSLP) was initially identified as being involved in lymphocyte development [1,2]. Subsequently, it was implicated in the induction of the pro-allergic phenotype in CD4+ effector T cells (Teff) [3]. Even though most studies to date have focused on the indirect mediation of allergic responses of TSLP via dendritic cells [4], it has been suggested that TSLP could directly expand CD4+ and CD8+ Teff [5, 6[. Recent studies revealed that TSLP could directly drive allergic responses of CD4+ Teff [7]. Studies of experimental models of asthma also indicated that TSLP-R-deficient animals failed to develop airway inflammation [4,8]. Conversely, over-expression of TSLP appeared to aggravate asthma symptoms [9]. Altogether, these evidences strongly suggested TSLP as a positive modulator of Th-2-biased inflammation.

Regulatory T cells (Treg) have emerged as a key regulator of inflammatory responses in allergic disorders [10,11]. Treg are CD4+CD25hiCD127lo/-Foxp3+ cells that possess suppressive activities against cytokine production and proliferation of Teff [12]. In studies of human allergic asthma (AA), decreased frequency and diminished suppressive activity of pulmonary Treg have been documented [13]. Furthermore, murine data suggested that Treg-mediated suppression reversed airway hyper-responsiveness, inflammation, and remodeling [14]. Immuno-suppressive cytokines such as IL-10 and TGF-β have been implicated in immune regulation by Treg during airway inflammation. For instance, co-expression of IL-10 and TGF-β by Treg allowed complete inhibition of airway hyper-reactivity [15]. In addition, suppression of Der-p1 and Mycobacterium vaccae-induced airway inflammation was dependent on IL-10 and/or TGF-β production by Foxp3+ Treg [16,17]. Therefore, modulation of the expression of these effector molecules by pulmonary Treg might play a critical role in regulating airway immune responses.

TSLP has been implicated in the development of Treg [18]. Disruption of TSLP signaling by TLSP receptor deletion impaired intra-thymic generation of Treg but did not affect their peripheral repertoire [19]. Consistent with these results, blocking TSLP-R led to a delayed functional maturation of thymic Treg [20]. Since TSLP and Treg have been suggested to play opposite modulatory roles in allergic inflammation, we aimed to explore the potential regulatory interaction between TSLP and Treg. Here we provided data that link TSLP signaling to the inhibition of Treg function as well as its implications in the context of peripheral tolerance in AA.

## Methods

### Human subjects

The study was approved by the Stanford Administrative Panel on Human Subjects in Medical Research. Study population included 15 AA subjects, 15 HC subjects, and 15 NA subjects. All subjects signed informed consent forms before participating in the study. Asthma diagnosis was established by evidences of episodic and partially reversible airflow obstruction or airway hyper-responsiveness, and exclusion of alternative diagnoses (NHLBI Expert Panel Report-3 2007). All patients had been followed for at least 6 months by a board certified Asthma, Allergy, and Immunology specialist at Stanford to ensure the diagnosis was correct. AA subjects were distinguished from NA subjects based on history of allergic symptoms, elevated blood IgE levels (above 50 IU/ml), and positive skin tests to allergens. Spirometry was performed by a fully qualified respiratory therapist and study coordinator, both of whom have over 15 years of experiences in asthma studies. Patients with FEV1 below 60% were considered severe. Those in the range of 60%-80% were considered moderate and those with FEV1 above 80% were considered mild. Comprehensive clinical data were collected at each patient visit including history, disease severity, medication status, common allergens, IgE level, and FEV1 (Additional file 1, Table S1). HC were defined as non-smoking subjects greater than 17 years of age with a total serum IgE of less than 25 IU/ml, negative skin testing (as compared to positive histamine control), and no evidence of lung disease or allergic symptoms. In addition, there was no evidence of obstructive or restrictive lung disease for HC on spirometry testing.

### Cell isolation

BAL samples were collected with a standardized protocol for clinical research at Lucile Packard's Children Hospital. After being collected, BAL samples (approximately 3 ml for each subject) were spun down at 1800 rpm for 15 minutes. Undiluted BAL supernatants were collected and filtered with 45 μm filters (BD Biosciences) and stored at -80°C until analysis. Cell pellets were subjected to downstream isolation. Untouched CD3+ T cells from BAL were first isolated by depleting B cells, monocytes/dendritic cells, NK cells, and granulocytes with pan T cell selection kit II (Miltenyi). CD4+ T cells were then isolated from these CD3+ T cells with CD4-microbeads (Miltenyi). Circulating CD4+ T cells were isolated from peripheral blood by CD4+ T cell Rosette kit (Stemcell) to deplete other cell lineages including B cells, monocytes/dendritic cells, NK cells, granulocytes, and non CD4+ T cells. Purified CD4+ T cell fraction, which contained virtually no DR+ antigen presenting cells (data not shown), was stained with CD25 (BD Biosciences) and CD127 (Biolegend) antibodies and sorted for CD4+CD25hiCD127lo/- Treg and Teff. Cells were rested in RPMI + 10% FBS + 1% L-Glutamine (complete media) after isolation. All procedures were performed with manufacturers' standard protocols.

### Cell stimulation

For cytokine priming experiments, cells were cultured at 1*10^5 ^cells per ml for 18 hours at 37°C in complete media in the presence or absence of 50 pg/ml recombinant IL-2, IL-7, and TSLP (Peprotech). For BAL incubation, pulmonary Treg from the same HC subject were cultured at 1*10^5 ^cells per 900 μl for 18 hours at 37°C in complete media in the presence of BAL from different AA and NA subjects. 100 μL of BAL supernatants were added to 900 μl of cell suspension. To determine the role of IL-10 in Treg-mediated suppression, recombinant IL-10 (Peprotech) or IL-10-blocking antibody (R&D) was added to suppression assays at different doses. Optimal volumes of BAL and cytokine/blocking antibody concentrations were experimentally determined. For neutralization experiments, blocking TSLP-R and isotype control antibody (R&D) were introduced to cell cultures at 10 μg/ml for 0.5 hours at 37°C before BAL supernatants were added. Optimal concentrations of blocking reagents were experimentally determined or used at doses recommended by the manufacturers.

### Immune phenotyping via FACS and ELISA

Phenotypes of immune cells were detected with antibodies against CD3, CD4, CD25, CD127, Foxp3, CTLA-4, LAG-3, OX40, and CD40L (Biolegend). For cytokine stimulation, Treg and Teff were cultured at 1*10^5 ^cells per ml for 2 hours after isolation. After 2 hours, 1 ml of cell suspension was stimulated with 50 ng/ml PMA (Sigma) and 1 μg/ml Ionomycin (Sigma) for 5 hours in the presence of Brefeldin A (diluted 1× solution was added in the last 2.5 hours, Biolegend) for intracellular cytokine staining or in the absence of Brefeldin A for ELISA. Staining for membrane-bound TGF-β was performed with standard surface staining protocols. For intracellular cytokine detection, stimulated cells were fixed and permeablized in 200 μL of Cytofix/Cytoperm solution (BD Biosciences) and then stained with antibodies against IL-4, IL-10, and TNF-α (Biolegend) in Permwash solution (BD Biosciences) in a final volume of 100 μL (BD Biosciences). Data acquisition threshold was set on forward scatter channel to exclude dead cells and debris with very low size. Compensation of flow cytometric data was performed electronically with Flow Jo (Treestar) for standardization. Quantitation of secreted cytokines was performed with ELISA kits for IL-4, IL-10, TGF-β (R&D), and TSLP (eBioscience). Total protein amount in BAL supernatants was determined by Bradford assays. TSLP level in BAL samples was normalized to total protein amount. All procedures were performed with manufacturers' standard protocols.

### Quantitation of TSLP-R mRNA

RNA was isolated using RNeasy kits (Qiagen) according to the manufacturer's protocol. Similar cell numbers (1*10^5 ^for peripheral blood cells and 5*10^4 ^for BAL cells) were used for each subject. For cDNA synthesis, 500 ng total RNA was transcribed with cDNA transcription reagents (Applied Biosystems) using random hexamers, according to the manufacturer's protocol. Gene expression was measured in real-time using primers and other reagents purchased from Applied Biosystems and Superarray. All PCR assays were performed in triplicates. Data was presented as relative fold expression of TSLP-R to the expression of the housekeeping gene β2-microglobulin.

### Detection of phosphorylated signal transducer and activator of transcription 5 (pSTAT5)

T cells were cultured at 1*10^5 ^cells per ml in complete media at 37°C for 18 hours after isolation. After 18 hours, 1 μl recombinant IL-2, IL-7, and TSLP (Peprotech) were added to V-bottom 96-well plates and 100 μl of cells were added to these wells with cytokines (final cytokine concentrations were 50 pg/ml). Optimal concentration and stimulation duration were experimentally determined. For ELISA, cells were lysed after being stimulated for 15 minutes. Lysates were analyzed for pSTAT5 by phospho-ELISA kits (R&D). For phospho-flow cytometry, cells were stimulated for 15 minutes at 37°C before being fixed with 10 μL of 10% paraformaldehyde at 37°C. Fixed cells were washed with PBS and permeablized with ice-cold methanol for 10 minutes. Permeablized cells were washed again with PBS and stained with pSTAT5 antibody (BD Biosciences). All procedures were performed with manufacturers' standard protocols. JAK3 inhibitor (WHI-P131, Calbiochem) was used at optimal concentration (78 μM) recommended by the manufacturer.

### Suppression assays

Standard thymidine-based suppression assays were performed to analyze Treg function. Treg and Teff were cultured at 3,750 cells per well in complete media with allogeneic irradiated CD3-depleted peripheral blood mononuclear cells (antigen presenting cells or APC), at 37,500 cells per well (1:1:10 ratio). Assays with 1:4:10 ratio of Treg:Teff: APC were also performed. Anti-CD3 antibodies (clone UCHT1, BD) were pre-coated on U-bottom 96 well plates at 5 μg/ml overnight at 37°C before suppression assays were performed. Additional media was added so the final volume in each well was 200 μl. On day 6, cells were pulsed with 1 μCi thymidine (25 μl) per well and harvested on day 7 with a Tomtec cell harvester. Thymidine incorporation was determined using a 1450 microbeta Wallac Trilux liquid scintillation counter. Stimulation assays were set up similarly with allogeneic irradiated APC and only one type of T cells. All assays were performed in triplicates.

### Statistical analysis

All statistical procedures were performed with Prism software (GraphPad). Non-parametric statistical tests were used for analysis of cohorts with small sample sizes (15 or less). Differences with p < 0.05 were considered statistically significant.

## Results and Discussion

### Activated Treg express TSLP-R and directly respond to TSLP-mediated activation of STAT5

**Figure 1:**
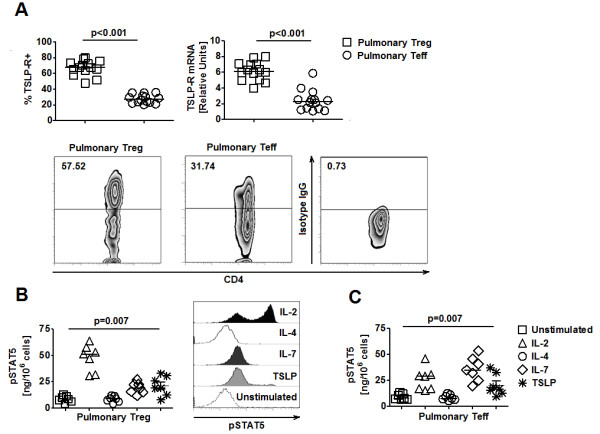
**Pulmonary Treg express functional TSLP-R**. **A**. *(Top) *TSLP-R expression at protein (% of positive cells) and mRNA levels in HC pulmonary Treg and Teff (n = 14). *(Bottom) *Representative FACS plots of TSLP-R expression by HC pulmonary Treg and Teff. **B**. *(Left) *Phosphorylated STAT5 (pSTAT5) expression, measured by ELISA, in HC pulmonary Treg in response to IL-2, IL-4, IL-7, and TSLP (n = 7). *(Right) *Representative FACS plots of pSTAT5 expression in HC pulmonary Treg in response to different stimuli. **C**. pSTAT5 expression, measured by ELISA, in HC pulmonary Teff in response to IL-2, IL-4, IL-7, and TSLP (n = 7). Wilcoxon tests were used for statistical analysis. Horizontal bars represented median values as indicated throughout the figure.

TSLP-R expression was first examined on purified CD3/CD28-activated pulmonary T cell subsets from healthy control (HC) subjects as described previously [5]. mRNA expression of TSLP-R was significantly higher in pulmonary Treg compared to pulmonary Teff (Figure 1A). Flow cytometry (FACS) analysis showed that, compared to pulmonary Teff, a significantly higher percentage of pulmonary Treg, express TSLP-R (Figure 1A). Consistent with these results, expression of TSLP-R positively correlated with CD25 expression and negative correlated with CD127 expression by tri-color FACS staining (Additional file 1, Figure S1A). TSLP signaling requires two receptor components, IL-7Rα and TSLP-R 21, the former of which was expressed at low level on Treg. Thus, to determine whether this pattern of high TSLP-R and low IL-7Rα expression was sufficient for TSLP signaling in Treg, we used phospho-ELISA, which allows measurement of protein expression in rare cell subsets, to quantify the expression of phosphorylated STAT5 (pSTAT5) by purified CD3/CD28-activated pulmonary Treg in response to recombinant TSLP. Our analysis showed that level of pSTAT5 in TSLP-stimulated pulmonary Treg was significantly elevated compared to that of un-stimulated cells (Figure 1B). The responsiveness of pulmonary Treg to TSLP was confirmed with phospho-flow cytometry (Figure 1B). While TSLP and IL-7 both signal via IL-7Rα, JAK3 phosphorylation was observed only in response to IL-7 [21-23]. Thus, signaling events triggered by binding of IL7 to IL-7Rα (the only signaling component in IL-7 receptor complex), but not binding of TSLP to TSLP-R (a part of TSLP receptor complex, which consists of two signaling components IL-7Rα and TSLP-R), resulted in JAK3 activation and subsequent induction of phosphorylated STAT5. To determine which receptor component (IL-7Rα vs. TSLP-R) in TSLP receptor complex was responsible for activation of STAT5 in response to TSLP, we introduced a JAK3 inhibitor into the stimulation assays. Phospho ELISA showed that in the presence of the JAK3 inhibitor, STAT5 activation was abrogated in response to IL-7 but not TSLP (Additional file 1, Figure S1B). These results suggested that signaling via TLSP-R, but not IL-7Rα, in response to TSLP was likely to be required for the induction of phosphorylated STAT5 in Treg. Consistent with previous findings 5, activated Teff could response to TSLP (Figure 1C). Similar results were also observed in circulating Treg and Teff (Additional file 1, Figure S2). Collectively, our results demonstrated that Treg expressed functional TSLP-R and directly responded to TSLP-mediated activation of STAT5.

### TSLP-primed Treg exhibit suboptimal suppressive activities

**Figure 2:**
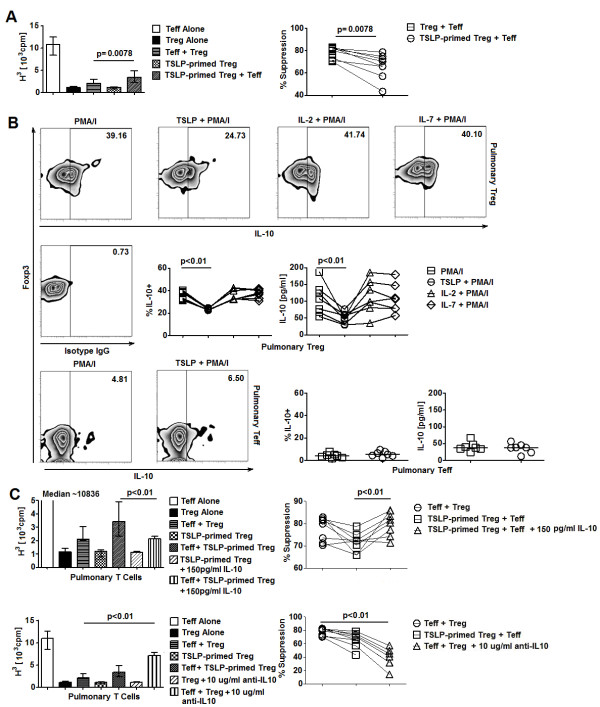
**TSLP inhibits suppressive activity and IL-10 production by Treg**. **A**. Suppressive activity of un-stimulated vs. TSLP-primed HC pulmonary Treg against Teff proliferation (n = 8) represented as thymidine counts in suppression assays *(left) *and percentage suppression of Teff proliferation *(right)*. Percentage suppression of Teff proliferation was calculated by percentage decrease in thymidine uptake in co-cultures of Teff and Treg compared to cultures of Teff alone. **B**. *(Top) *Representative FACS plots of IL-10 production by PMA/Ionomycin (PMA/I) activated HC pulmonary Treg after being primed with IL-2, IL-7, and TSLP. *(Middle) *Expression of IL-10 by PMA/I activated HC pulmonary Treg after being primed with different cytokines (n = 7). *(Bottom left) *Representative FACS plots of IL-10 production by PMA/Ionomycin (PMA/I) activated HC pulmonary Teff after being primed with TSLP. (*Bottom right) *Expression of IL-10 by PMA/I activated HC pulmonary Teff after being primed with TSLP (n = 7). Data represented intracellular flow cytometric and ELISA results. **C**. *(Top) *Effects of exogenous IL-10 on suppressive activity of TSLP-primed HC pulmonary Treg against Teff proliferation (n = 7). *(Bottom) *Effects of neutralizing antibodies against IL-10 on suppressive activity of HC pulmonary Treg against Teff proliferation (n = 7). Data were represented as thymidine uptake in suppression assay cultures *(left) *as well as percentage suppression of Teff proliferation *(right)*. Wilcoxon tests were used for statistical analysis. Bar graphs and horizontal bars represented median values as indicated throughout the figure.

Subsequently, *in vitro *stimulation assays were utilized to determine the effects of TSLP signaling in Treg. Purified CD3/CD28-activated HC pulmonary Treg were incubated with 50 pg/ml TSLP for 18 hours before being subjected to downstream assays. TSLP-primed pulmonary Treg did not exhibit increased proliferation (Additional file 1, Figure S3A). Expression of surface and intracellular molecules associated with suppressive function of pulmonary Treg such as LAG-3, CTLA-4, and Foxp3 was not significantly altered after exposure to TSLP (Additional file 1, Figure S4). CD40L and OX40, molecules that have been associated with TSLP-mediated induction of pro-inflammatory cytokine production and inhibition of IL-10 production by T cells [24,25] were expressed at similar levels between TSLP-primed and un-stimulated pulmonary Treg (Additional file 1, Figure S4). To further characterize the effects of TSLP on Treg function, we performed *in vitro *suppression assays to assess the ability of Treg to inhibit Teff proliferation after they have been exposed to TSLP. CD3/CD28-activated Treg were pre-incubated with TSLP for 18 hours and underwent to 3 washes to remove TSLP in the pre-incubation cultures before subjected to suppression assays. We found decreased suppressive activities of TSLP-primed pulmonary Treg compared to cultures with un-stimulated pulmonary Treg (Figure 2A). A similar effect of TSLP was observed in circulating T cells (Additional file 1, Figure S3B, C). Since Treg exert their suppressive activity via cell contact as well as release of suppressive molecules [26-29], we tested whether the inhibitory effects of TSLP occurred via the former mechanism with transwell assays. Treg were able to suppress Teff proliferation in the presence of transwell inserts even though their suppression potency decreased (Additional file 1, Figure S3D). Interestingly, decrease in suppressive activities of TSLP-primed Treg was also observed in the presence of transwell inserts (Additional file 1, Figure S3D). Thus, suppressive activity of pulmonary Treg was significantly reduced by exposure to TSLP. This effect did not occur via cell-contact dependent suppressive mechanisms and was likely to be mediated via TSLP-mediated inhibition of soluble factors produced by Treg. These results were consistent with our previous findings which showed that TSLP did not alter the expression of LAG-3 and CTLA-4, molecules that are involved in cell-contact dependent suppressive activities of Treg [30].

### TSLP suppresses IL-10 production by Treg

We next explored the influence of TSLP on Treg-derived soluble mediators and their potential association with the TSLP-induced impairment of Treg function. Purified CD3/CD28-activated pulmonary Treg were incubated with 50 pg/ml TSLP for 18 hours before cytokine detection. Intracellular staining showed that TSLP-primed pulmonary Treg exhibited a significant reduction in IL-10 expression compared to those that were not exposed to TSLP (Figure 2B). A similar reduction in IL-10 expression by TSLP-primed pulmonary Treg was confirmed via ELISA (Figure 2B). Surprisingly, this effect was only present in the pulmonary Treg subset as similar TSLP-priming experiments of pulmonary Teff showed no significant changes in IL-10 expression by these cells (Figure 2B). Furthermore, no inhibitory effects of TSLP on the production of immunosuppressive cytokines TGF-β expression by pulmonary Treg was not observed (Additional file 1, Figure S5A). TSLP also did not enhance the production of pro-inflammatory cytokines IL-4 and TNF-α in pulmonary Treg and Teff (Additional file 1, Figure S5A, B). We also examined the priming effects of TSLP on circulating T cell subsets and found that TLSP was also able to suppress their IL-10 production (Additional file 1, Figure S6). Consistent with our findings on pulmonary cells, this inhibitory effect of TSLP on IL-10 production was specific to circulating Treg but not Teff (Additional file 1, Figure S6). Altogether, these findings suggested that TSLP directly inhibited IL-10 production by human Treg.

To determine whether IL-10 inhibition was involved in TSLP-induced impairment of Treg function, we attempted to rescue the reduced suppression of Teff proliferation mediated by TSLP-primed Treg with exogenous IL-10. Addition of recombinant IL-10 to suppression assays with TSLP-primed Treg significantly reduced thymidine uptakes in these cultures (Figure 2C, Additional file 1, Figure S7A). Conversely, blocking IL-10 by neutralizing antibodies in suppression assays with Treg that were not exposed to TSLP significantly increased cell proliferation (Figure 2C, Additional file 1, Figure S7B). Altogether, these results demonstrated that TSLP-mediated inhibition of Treg function might be contributed by their suppressive effects on IL-10 production of Treg.

### Reduced function of allergic asthmatic pulmonary Treg was associated with elevated airway TSLP

**Figure 3:**
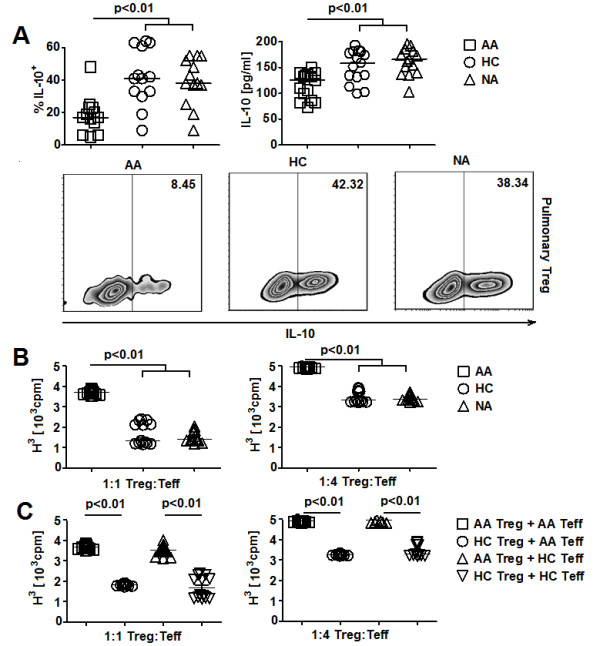
**Decreased IL-10 production and suppressive function of allergic asthmatic pulmonary Treg**. **A**. *(Top) *IL-10 expression by PMA/I activated pulmonary Treg from AA, HC, and NA subjects (n = 13). Data represented intracellular flow cytometric and ELISA results. *(Bottom) *Representative FACS plots of IL-10 expression by pulmonary Treg from different subject groups. **B**. Suppressive activity of pulmonary Treg against autologous Teff from AA, HC, and NA subjects represented as thymidine counts in suppression assays (n = 13). **C**. Allogeneic suppression assays with pulmonary T cells from AA and HC subjects (n = 7). Treg and Teff were cultured at 1:1 and 1:4 ratios. Kruskal Wallis tests (A, B) and Wilcoxon tests (C) were used for statistical analysis. Horizontal bars represented median values as indicated throughout the figure.

To explore the potentially pathological role of TSLP-mediated inhibition of Treg function, we next examined the interaction between TSLP and Treg in allergic asthma. Elevated TSLP expression has been observed in bronchoaveolar lavage fluid (BAL) of allergic asthmatic (AA) subjects [31,32]. Thus, it is likely that AA pulmonary Treg function might be influenced by elevated expression of BAL TSLP. Pulmonary Treg from HC, AA, and non-allergic asthmatic (NA) subjects were purified and subjected to *in vitro *functional assays (Additional file 1, Figure S8A). AA pulmonary Treg activated with PMA and Ionomycin showed a significant decrease in IL-10 expression compared to HC and NA counterparts (Figure 3A). No significant changes in IL-10, TNF-α production by pulmonary Teff; and IL-4, TNF-α, and TGF-β expression by pulmonary Treg were observed among 3 subject groups (Additional file 1, Figure S8B). We also observed an increase in cell proliferation in suppression assays with AA pulmonary Treg and autologous Teff compared to assays with HC and NA cells (Figure 3B). Furthermore, in allogeneic suppression assays, AA pulmonary Treg showed a significant decrease in suppressive activity against HC pulmonary Teff compared to HC pulmonary Treg (Figure 3C). On the other hand, HC pulmonary Treg suppressed the proliferation of AA and HC pulmonary Teff equivalently (Figure 3C). Collectively, these results suggested the presence of defective IL-10 production and suppressive function by AA pulmonary Treg.

### Elevated expression of TSLP in AA BAL is necessary for its suppressive effects on Treg function

**Figure 4:**
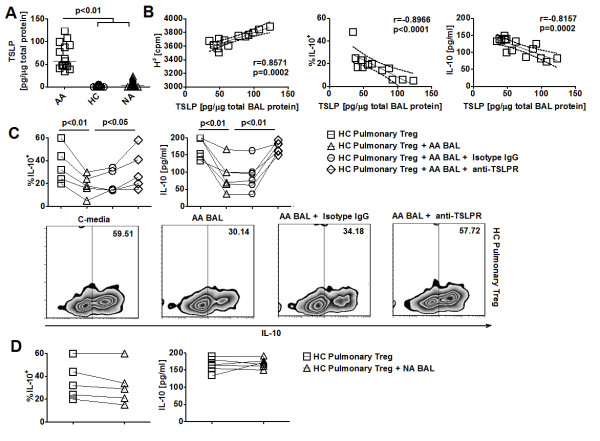
**Impaired allergic asthmatic pulmonary Treg function is associated with elevated airway TSLP**. **A**. TSLP expression in BAL of AA, HC, and NA subjects (n = 15). **B**. Correlation of TLSP levels in BAL of AA subjects with IL-10 production and suppression activity of AA pulmonary Treg (n = 13). Straight lines represented best-fitted lines. Dotted lines represented 95% confident intervals. **C**. *(Top) *Effects of blocking TLSP-R antibodies on AA BAL-mediated inhibition of IL-10 production by HC pulmonary Treg (n = 5). *(Bottom) *Representative FACS plots of IL-10 production by HC pulmonary Treg in different stimulatory conditions. **D**. Effects of NA BAL on IL-10 production by HC pulmonary Treg (n = 5). Data represented intracellular flow cytometric and ELISA results. Kruskal Wallis tests (A), Spearman tests (B) and Wilcoxon tests (C) were used for statistical analysis. Horizontal bars represented median values as indicated throughout the figure.

Consistent with previous findings, we found elevated expression of TSLP in AA BAL (Figure 4A). Elevated BAL TSLP expression was significantly correlated with reduced IL-10 expression and suppressive function of pulmonary AA Treg (Figure 4B). These results thus suggested that the selective dysfunction in IL-10 production by pulmonary Treg in AA might be associated with *in vivo *priming of these cells by TSLP. We next examined whether BAL from AA subjects with high concentrations of TSLP could induce functional changes in HC pulmonary Treg as previously seen with recombinant TSLP. BAL samples from AA subjects with highest levels of TSLP were selected for this experiment. BAL supernatants were introduced to CD3/CD28 activated HC pulmonary Treg cultures at a 1:10 volume ratio for 18 hours and Treg were subsequently analyzed for IL-10 production. Surprisingly, we found that HC pulmonary Treg incubated with AA BAL showed significantly decreased expression of IL-10 (Figure 4C). In contrast, in similar priming experiments, BAL samples from NA subjects failed to inhibit IL-10 production by HC pulmonary Treg (Figure 4D). Furthermore, this reduction in IL-10 expression by HC pulmonary Treg mediated by AA BAL was reversed by neutralizing TSLP with 10 μg/ml of a blocking antibody against TSLP-R (Figure 4C). On the other hand, parallel cultures with low-endotoxin isotype control antibodies at a similar concentration failed to reverse the inhibition of IL-10 production of Treg by AA BAL (Figure 4C). Altogether, these results showed that IL-10 production by pulmonary HC Treg could be inhibited by exposure to AA-airway-derived-TSLP.

It has been well established that TSLP is a master regulator of airway inflammation because of its abundant expression in airway epithelial cells as well as its ability to instruct antigen presenting cells to prime the development of pathogenic Th-2 helper T cells. Here we showed that functional TSLP-R was expressed on Treg from both blood and pulmonary compartments. Furthermore, TSLP directly activated the signal-transducing molecule STAT5 by Treg and suppressed their suppressive activities and production of the immunosuppressive cytokine IL-10. Our results thus pointed to a potentially novel mechanism by which TSLP might directly dampen tolerogenic immune responses of Treg, and subsequently prolong the course of inflammation.

TSLP signaling pathway resembles that of a cytokine family, including IL-2, IL-7, and IL-15, which all activates STAT5. Surprisingly, unlike TSLP, cytokines such as IL-2 and IL-15 have been reported to enhance IL-10 production by Treg [33,34]. Thus, distinctions in signaling pathways downstream of TSLP-R-TSLP ligation with respect to the activation of intracellular signaling molecules other than STAT5 might be required for the inhibition of Treg function by TSLP. In our study, the inhibitory effect of TSLP on IL-10 production was specific to Treg but not Teff. This result might be explained by potential differences in signaling circuitries between Treg and Teff, which have been observed in AKT/mTOR cascade [35]. Proteomic analysis of intracellular signaling molecules in Treg and Teff is underway in our laboratory to provide further insights to this phenomenon.

Contrary to a previous report which showed that TSLP could enhance IL-4 production by Teff isolated from allergen-sensitized mice [7], we did not find a modulatory role of TSLP on IL-4 production by Teff. It is worth noting that antigen presenting cells, which include monocytes and dendritic cells, were depleted in our culture system while present in theirs. Therefore, it was possible that the ability of TSLP to stimulate IL-4 production by Teff required cross-talk between dendritic cells and TSLP-primed Teff even in the absence of direct TSLP-dendritic cell interaction. In addition, Jiang et al showed that blocking TSLP reduced thymic Foxp3 expression [36]. However, we did not find a modulatory role of TSLP on the expression of Foxp3 by peripheral Treg. Thjs discrepancy might be due to changes in microenvironments in the thymus vs. peripheral tissues with respect to the presence of different cellular subsets and cytokines, which may have an impact on Treg homeostasis. Alternatively, modulation of Foxp3 expression in Treg by TSLP might be temporally regulated: naïve thymic derived Treg, but not circulating/tissue resident Treg, might be susceptible to TSLP-mediated up-regulation of Foxp3.

Our study also showed an association between TSLP-Treg interaction and defective function of pulmonary Treg in human AA. Pulmonary Treg showed decreased IL-10 production, which was correlated with the increased expression of TSLP in BAL of these subjects. Consistent with the potential role of TSLP in the suppression of IL-10 by pulmonary AA Treg, we showed that TSLP derived from BAL of AA subjects was necessary for the direct suppression of IL-10 production by HC Treg *in vitro*. Alternatively, decreased IL-10 production might result from *in vivo *exposure of AA Treg to different stimuli in AA airway and interaction among TSLP, pulmonary dendritic cells, and pulmonary Treg, respectively. A previous study showed that defective IL-10 production was observed on steroid-resistant asthmatic subjects which could be pharmacologically reversed by calcitriol [37]. In this study, the population of regulatory T cells being examined was IL-10 producing CD4+ T cells (Tr1). Thus, it is not known whether IL-10 production by natural Foxp3-expressing Treg is also defective. Our preliminary studies showed that steroid-resistant asthmatics also had elevated TSLP levels in their BAL (unpublished observations), suggesting that this increased expression of TSLP might also have an inhibitory effect on expression of IL-10 by natural Treg. Studies to elaborate on these findings as well as to characterize the role of TSLP on IL-10 producing CD4+ T cells (Tr1) and the ability of calcitriol to reverse these effects of TSLP on Treg production of IL-10 are underway in our laboratory.

Defective IL-10 production by pulmonary Treg was found only in AA but not NA subjects. These results were consistent with previous observations of immunological differences in these two sub-types of asthma [38]. Nevertheless, our data did not rule out the possibility that pulmonary Treg exhibit distinct suboptimal regulatory functions in other allergic and pulmonary diseases. A potential explanation is that each disease possesses a signature inflammatory environment. Thus, the effects of inflammatory cytokine milieu of each disease on pulmonary Treg function might very well be different.

Modulation of TSLP signaling has been shown to be influential to airway inflammation in experimental models of asthma [39]. Blockade of TSLP-R suppressed allergic inflammation by altering dendritic cell function. Deletion of an intracellular regulator of TSLP production, SOCS7, also led to increased allergic phenotype [40]. Along with these results, our data suggest that, besides preventing airway inflammation, TSLP-targeted therapies might also be beneficial in modulating immune tolerance by Treg in allergic diseases.

## Conclusions

Our study presents a potentially novel antigen-presenting-cell-independent mechanism by which TSLP might contribute to the exacerbation of airway inflammation by directly inhibiting tolerogenic immune responses of pulmonary Treg.

## Abbreviations

HC: healthy controls; AA: allergic asthmatics; NA: non-allergic asthmatics; Treg: CD4+CD25hiCD127lo/-regulatory T cells; Teff: CD4+CD25-T cells; TSLP: thymic stromal lymphopoietin; pSTAT5: phosphorylated signal transducer and activator of transcription 5; BAL: bronchoaveolar lavage fluid; PBMC: peripheral blood mononuclear cells; FEV1: forced expiratory volume in 1 second; FACS: fluorescent activated cell sorting; MFI: mean fluorescence intensity.

## Competing interests

The authors declare that they have no competing interests.

## Authors' contributions

KDN designed the study and wrote the article. KCN oversaw the project and recruited study subjects. KDN, KCN, and CV performed experiments and analyzed the data. All authors read and approved the final manuscript.

## Supplementary Material

Additional file 1 **Supplementary Figures and Table**. The file includes Figures S1-8 and Table S1. Click here for file
