# Supplementary material for: TSLP directly impairs pulmonary Treg function: association with aberrant tolerogenic immunity in asthmatic airway
Source: Allergy Asthma Clin Immunol. 2010 Mar 15;6(1):4. doi: 10.1186/1710-1492-6-4 (PMC3161393; doi:10.1186/1710-1492-6-4)
Supplement: Additional file 1 — Supplementary Figures and Table. The file includes Figures S1-8 and Table S1. [file 1710-1492-6-4-S1.DOC]

**Additional File 1**

**Title:** Supplementary Figures and Table.

**Description:**  The file includes Figures S1-8 and Table S1.

**Figure S1. Pulmonary Treg express functional TSLP-R. A.** *(Left)* Gating strategy of pulmonary CD4+CD25+CD127lo/- Treg from total CD4+ T cells. *(Middle)* Co-staining of CD25 vs. TSLP-R and CD127 vs. TSLP-R in CD4+ T cells. *(Right)* Expression of TSLP-R in myeloid dendritic cells. **B.** *(Left)* Expression of pSTAT5 in pulmonary Treg measured by ELISA in response to recombinant TSLP and IL-7 in the presence or absence of a JAK kinase inhibitor (JAK Inh) (n=7). *(Right)* Representative FACS plot of pSTAT5 expression in pulmonary Treg in response to recombinant TSLP and IL-7 in the presence or absence of a JAK kinase inhibitor. Wilcoxon tests were used for statistical analysis. Bar graphs and horizontal bars represented median values as indicated throughout the figure.

**Figure S2. Circulating Treg express functional TSLP-R. A.** TSLP-R expression at protein *(left)* and mRNA *(right)* levels in HC circulating Treg and Teff (n=11). **B.** Comparison of pSTAT5 expression between HC circulating Treg and Teff in response to TSLP (n=7). Wilcoxon tests were used for statistical analysis. Bar graphs and horizontal bars represented median values as indicated throughout the figure.

**Figure S3. Characterization of TLSP effects on pulmonary and circulating T cells. A.** Proliferation of un-stimulated vs. TSLP-primed HC pulmonary Treg in response to recombinant IL-2 (n=7). **B.** Proliferation of un-stimulated vs. TSLP-primed HC circulating Treg in response to recombinant IL-2 (n=7). **C.** Suppressive activity of un-stimulated vs. TSLP-primed HC circulating Treg against Teff proliferation (n=8). **D.** Suppressive activity of un-stimulated vs. TSLP-primed HC pulmonary Treg against Teff proliferation in the present of transwell inserts (n=8). Wilcoxon tests were used for statistical analysis. Bar graphs and horizontal bars represented median values as indicated throughout the figure.

**Figure S4. Phenotype of TSLP-primed pulmonary Treg. A.** Comparison of Foxp3, LAG-3, CTLA-4, OX40, and CD40L expression between un-stimulated vs. TSLP-primed HC pulmonary Treg (n=7). **B.** Representative FACS plots of Foxp3, LAG-3, CTLA-4, OX40, and CD40L expression in un-stimulated vs. TSLP-primed pulmonary Treg. Horizontal bars represented median values as indicated throughout the figure.

**Figure S5. Cytokine prolife of TSLP-primed pulmonary T cells. A.** *(Left)* Representative FACS plots of TGF-β, TNF-α, and IL-4 production in un-stimulated vs. TSLP-primed HC pulmonary Treg. *(Right)* Comparison of TGF-β, TNF-α, and IL-4 production between un-stimulated vs. TSLP-primed pulmonary Treg (n=7). **B.** *(Left)* Representative FACS plots of TNF-α and IL-4 production in un-stimulated vs. TSLP-primed HC pulmonary Teff. *(Right)* Comparison of TNF-α and IL-4 production between un-stimulated vs. TSLP-primed HC pulmonary Teff (n=7). Data represented intracellular flow cytometric and ELISA results. Horizontal bars represented median values as indicated throughout the figure.

**Figure S6. Cytokine prolife of TSLP-primed circulating T cells.** Comparison of IL-10 production between un-stimulated vs. TSLP-primed HC circulating Treg *(top)* and Teff *(bottom)* (n=7). Data represented intracellular flow cytometric and ELISA results. Wilcoxon tests were used for statistical analysis. Horizontal bars represented median values as indicated throughout the figure.

**Figure S7. Influence of IL-10 in Treg-mediated suppression assays. A.** Effects of exogenous IL-10 on suppressive activity of TSLP-primed HC pulmonary *(top)* and circulating *(bottom)* Treg (n=8). **B.** Effects of neutralizing antibodies against IL-10 on suppressive activity of HC pulmonary *(top)* and circulating *(bottom)* Treg (n=8). Data were represented as thymidine uptake in suppression assay cultures *(left)* as well as percentage suppression of Teff proliferation *(right)*. Friedman tests were used for statistical analysis. Bar graphs represented median values as indicated throughout the figure.

**Figure S8. Foxp3 expression and cytokine profile of pulmonary T cells among different subject groups. A.** Representative FACS plots of Foxp3 and CD25 expression in purified pulmonary Treg among different subject groups. **B.** TGF-β, IL-4, and TNF-α production by pulmonary Treg among different subject groups (n=12). **C.** IL-10, IL-4, and TNF-α production by pulmonary Teff among different subject groups (n=12). Data represented ELISA results. Kruskal Wallis tests were used for statistical analysis. Bar graphs represented median values as indicated throughout the figure.

**Table S1. Subject demographics.**

**Figure S1**

**
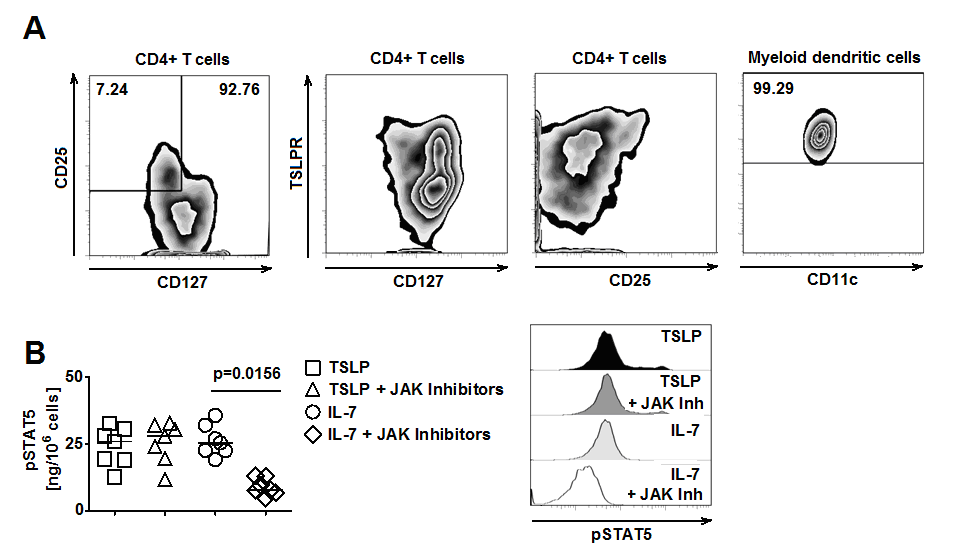
**

**Figure S2**

**
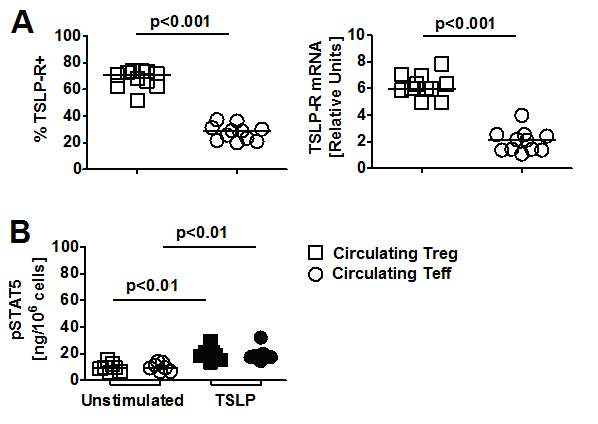
**

**Figure S3**

**
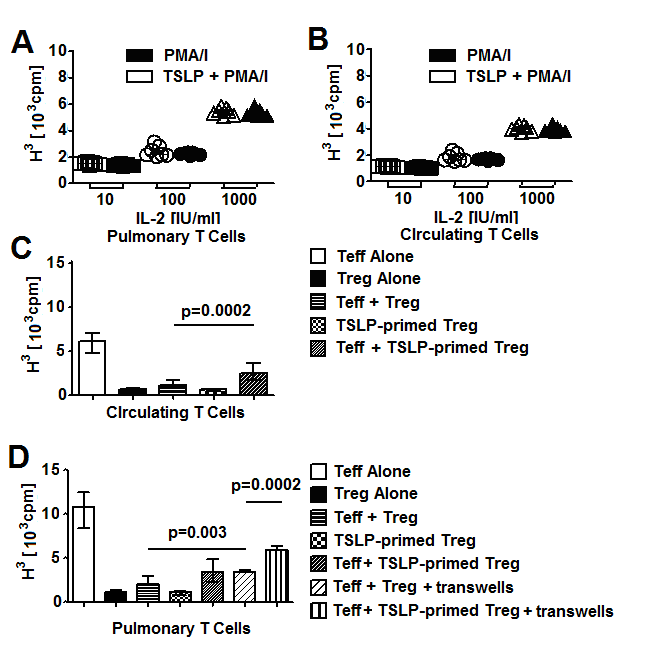
**

**Figure S4**


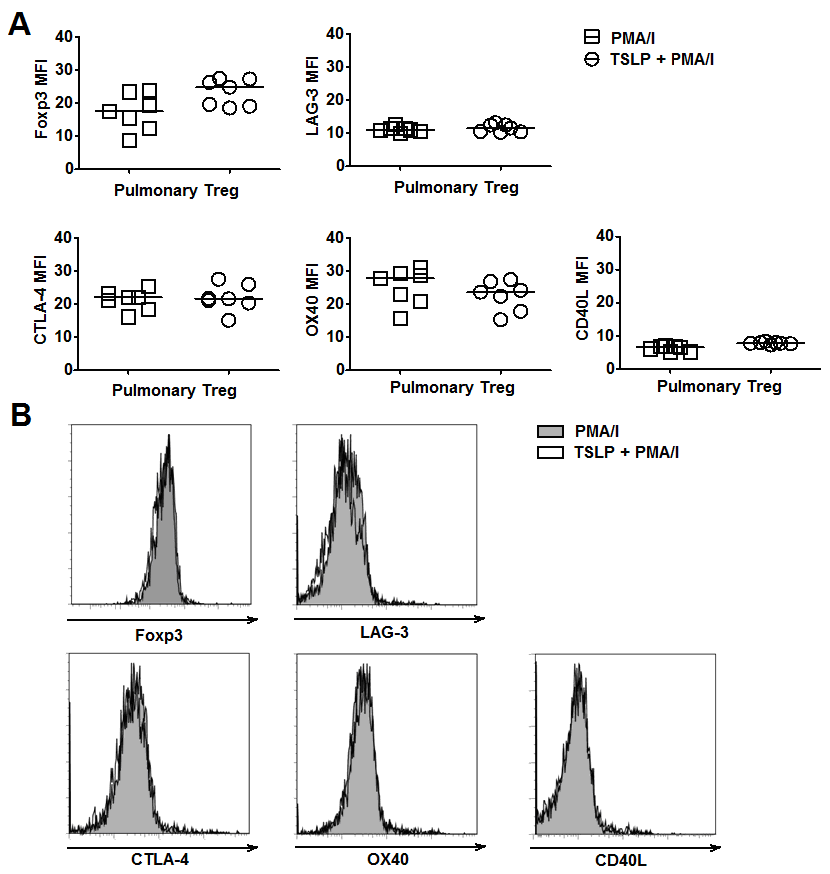


**Figure S5**

**
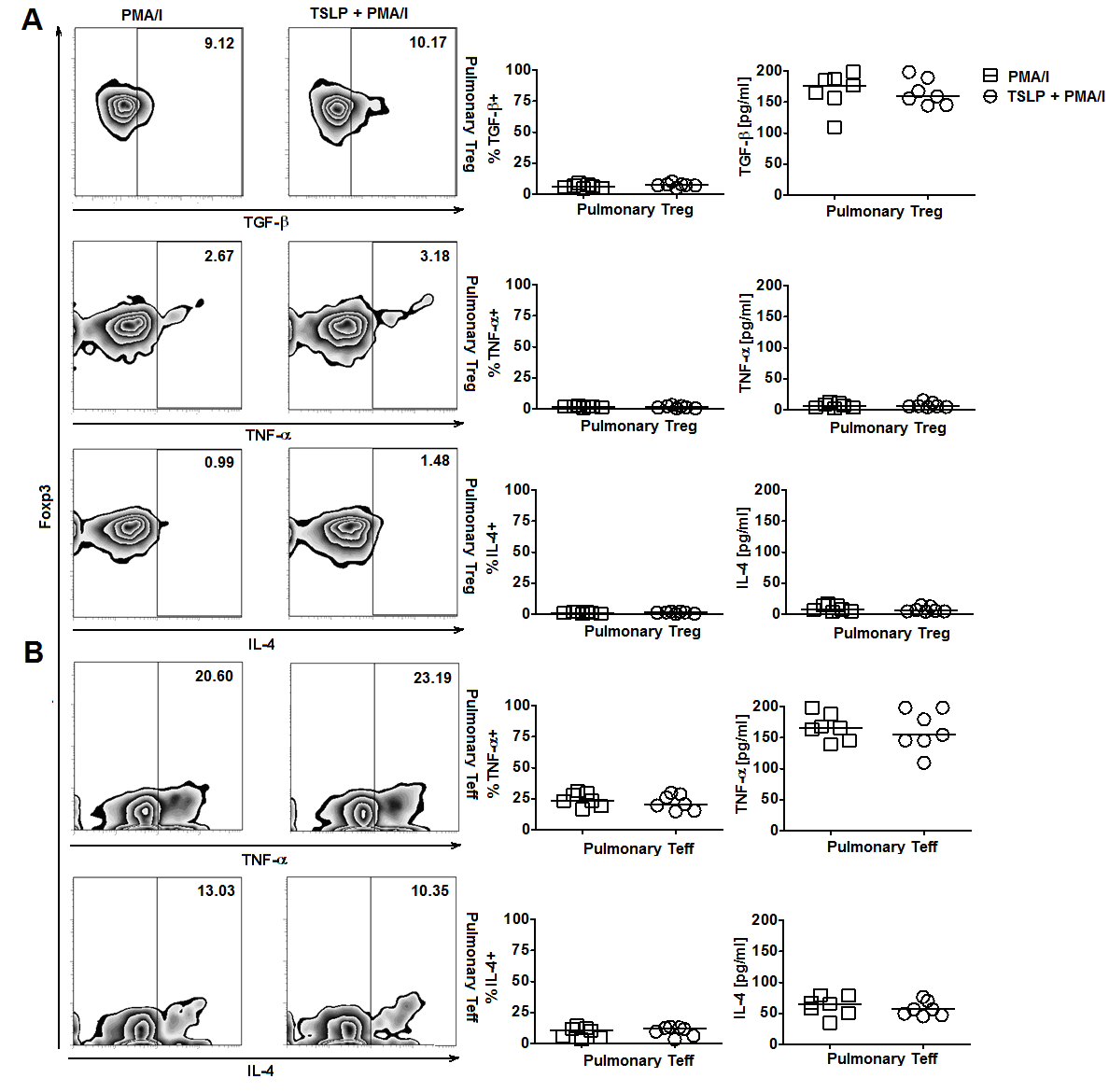
**

**Figure S6**

**
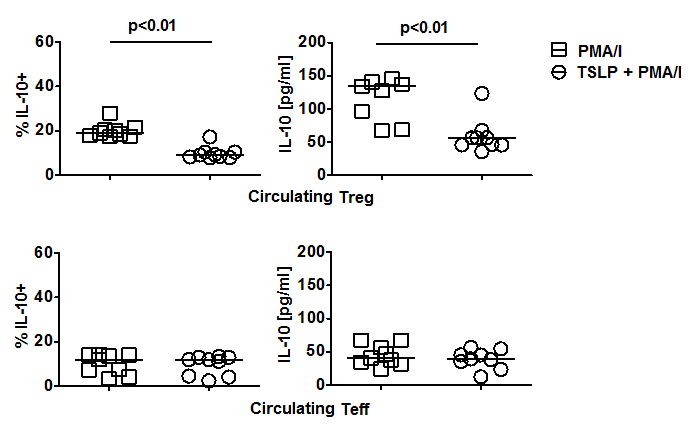
**

**Figure S7**

**
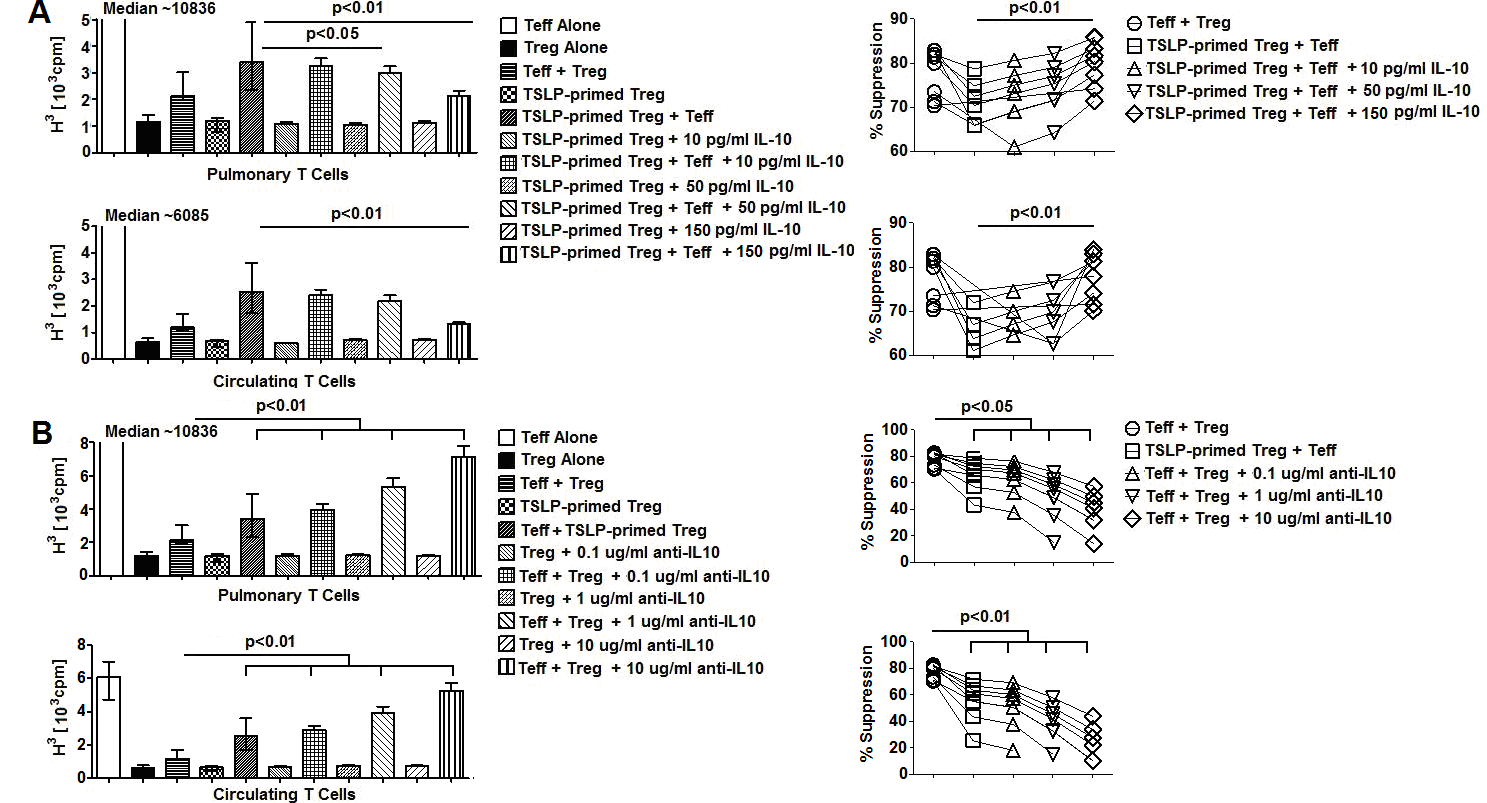
**

**Figure S8**

**
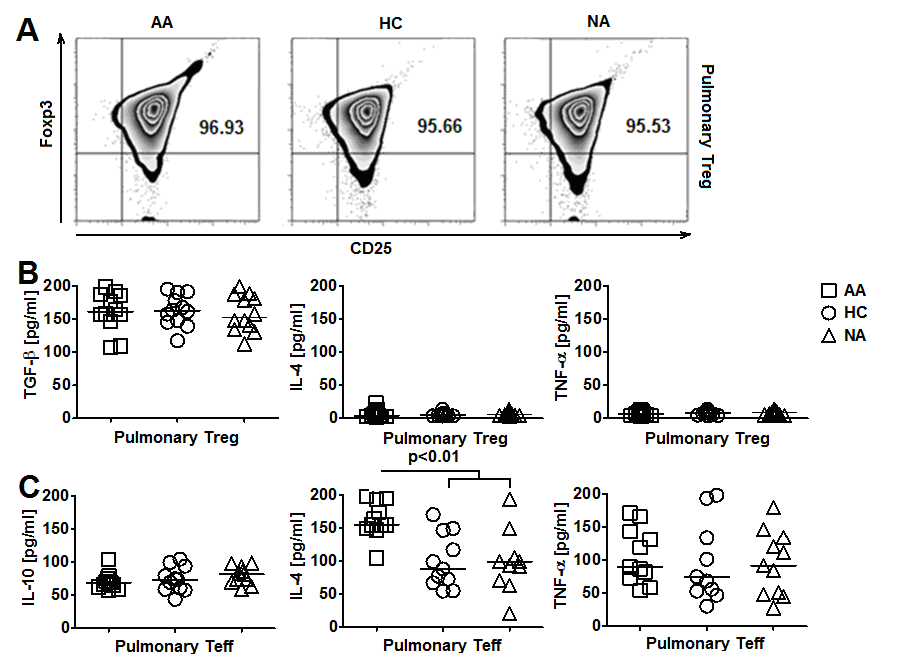
**

**Table S1**

***
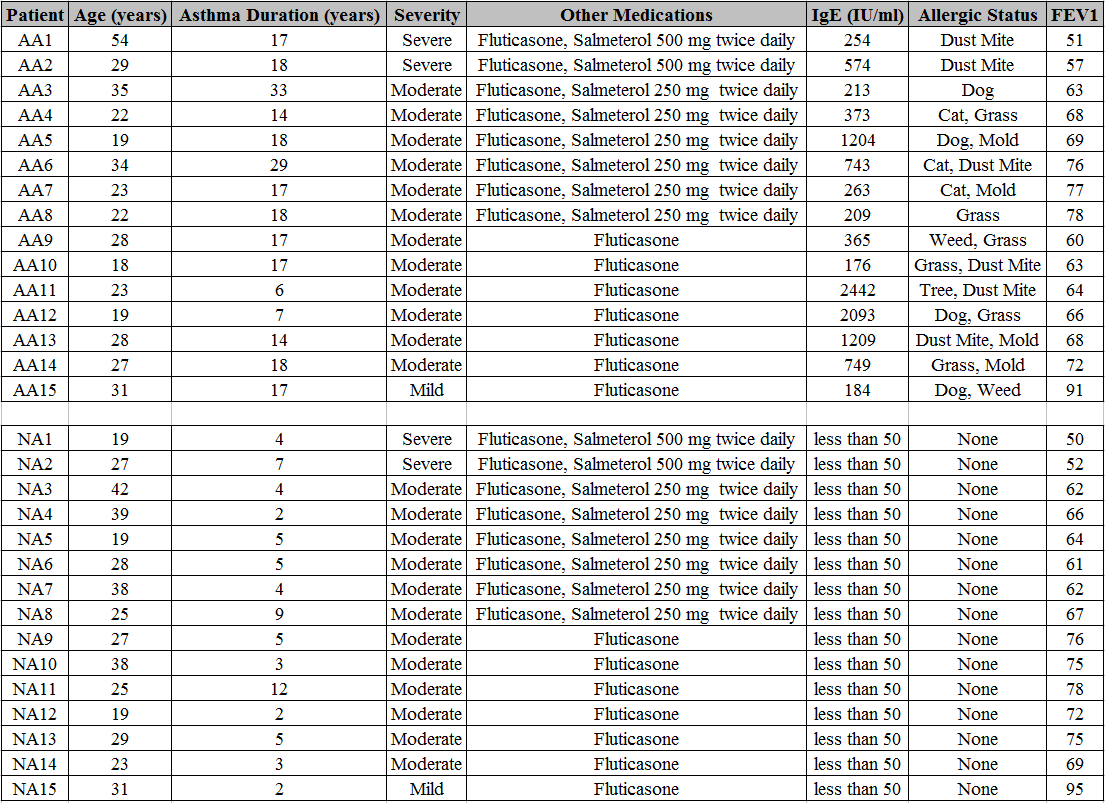
***
